# IL4 induces IL6-producing M2 macrophages associated to inhibition of neuroinflammation in vitro and in vivo

**DOI:** 10.1186/s12974-016-0596-5

**Published:** 2016-06-07

**Authors:** Giacomo Casella, Livia Garzetti, Alberto T. Gatta, Annamaria Finardi, Chiara Maiorino, Francesca Ruffini, Gianvito Martino, Luca Muzio, Roberto Furlan

**Affiliations:** Clinical Neuroimmunology Unit, Institute of Experimental Neurology—INSpe, Division of Neuroscience, San Raffaele Scientific Institute, Via Olgettina 58, 20132 Milan, Italy; Neuroimmunology Unit, Institute of Experimental Neurology—INSpe, Division of Neuroscience, San Raffaele Scientific Institute, Milan, Italy

**Keywords:** IL4, IL6, M2 macrophages, Autoimmunity, EAE

## Abstract

**Background:**

Myeloid cells, such as macrophages and microglia, play a crucial role in neuroinflammation and have been recently identified as a novel therapeutic target, especially for chronic forms. The general aim would be to change the phenotype of myeloid cells from pro- to anti-inflammatory, favoring their tissue-trophic and regenerative functions. Myeloid cells, however, display a number of functional phenotypes, not immediately identifiable as pro- or anti-inflammatory, and associated to ambiguous markers.

**Methods:**

We employed in vitro assays to study macrophage polarization/differentiation in the presence of classical polarizing stimuli such as IFNγ (pro-inflammatory) and IL4 (anti-inflammatory). We induced neuroinflammation in mice by immunization with a myelin antigen and treated diseased mice with intracisternal delivery of an IL4-expressing lentiviral vector. We analyzed clinical, pathological, and immunological outcomes with a focus on myeloid cells.

**Results:**

We found that IL6, usually considered a pro-inflammatory cytokine, was released in vitro by macrophages treated with the anti-inflammatory cytokine IL4. We show the existence of macrophages expressing IL6 along with classical anti-inflammatory markers such as CD206 and demonstrate that these cells are immunosuppressive in vitro. In neuroinflamed mice, we show that IL4 delivery in the central nervous system (CNS) is associated with clinical and pathological protection from disease, associated with increased IL6 expression in infiltrating macrophages.

**Conclusions:**

IL6 is known to mediate both pro- and anti-inflammatory effects, having two distinct ways to induce cell-signaling: either through the membrane bound receptor (anti-inflammatory) or through trans-signaling (pro-inflammatory). We show here that IL6-expressing macrophages are associated to protection from neuroinflammation, suggesting that IL6 anti-inflammatory properties prevail in the CNS, and calling for a general reconsideration of IL6 in macrophage polarization.

**Electronic supplementary material:**

The online version of this article (doi:10.1186/s12974-016-0596-5) contains supplementary material, which is available to authorized users.

## Background

Plasticity and flexibility are key features of myeloid cells, like macrophages and microglia, and of their activation states. To categorize macrophage functions, currently, the M1/M2 paradigm is used [1, 2]. Generally, M1 refers to the classically activated macrophages, whereas M2 to the alternatively activated macrophages [3, 4]. Polarized macrophages differ in terms of receptor expression, cytokine production, effector functions, and chemokine repertoires [5]. M1 macrophages are differentiated by microbial products such as LPS, or by IFNγ produced during an adaptive immune response by TH1 cells or during an innate immune response by natural killer (NK) cells. M1 macrophages have microbicidal or tumoricidal capacity in host defense and express, among others, iNOS, IL1β, and TNFα. Alternatively activated macrophages differentiate in several subtypes induced by various stimuli: M2a induced by IL4 and IL-13, M2b induced by exposure to immune complex (IC), and agonist of toll-like receptors (TLRs) or IL-1R, and M2c induced by IL-10 and glucocorticoids [1, 6, 7]. M2 macrophages express IL12 at low levels but display high levels of scavenging, mannose, and galactose receptors, chitinase molecules such as YM1, arginase, and chemokines such as CCL17 and CCL22. The M1/M2 paradigm is an oversimplified model to define the functional phenotypes of phagocytes, and in vivo macrophages can adopt a wide array of phenotypes depending on the stimuli coming from the tissue microenvironment [2]. Markers currently adopted to define M1/M2 macrophages, however, are ambiguous, and several of them can be found on both phenotypes. One of those is IL6, classically associated to M1 but detected also in some M2 subtypes [1, 8, 9]. IL6 is a pleiotropic cytokine, displaying both pro- and anti-inflammatory activity [10]. IL6 pro-inflammatory activity descriptions prevail in literature, although its ability to promote M2 macrophage polarization has been recently reported [11]. Macrophages, along with microglia, are the main effector cells in experimental autoimmune encephalomyelitis (EAE), the mouse model for multiple sclerosis [12, 13]. In particular, macrophage and microglia polarizations during persistent neuroinflammation are considered a crucial event for the development of chronic phases of multiple sclerosis, still lacking any treatment.

We have extensively shown the therapeutic potential of IL4 CNS gene delivery in rodent and primate models of neuroinflammation [14–19]. In previous works, we have associated the protective effect of IL4 to the modulation of T helper lymphocytes, and especially to the increased recruitment of T regulatory cells [18].

We asked the question if cells of the innate immune system are also modulated and contribute to the therapeutic effect of IL4 delivery in neuroinflammation. We show here that IL4 is able to induce IL6 release from M2 macrophages in vitro. When delivered to the CNS during neuroinflammation, IL4 modulates macrophage polarization, and we found that IL6 was specifically associated to the protective myeloid phenotype.

## Methods

### Bone marrow (BM)-derived macrophages in vitro culture

Bone marrow (BM) cells were flushed from femur and tibia of C57BL/6 mice and cultured with macrophage colony-stimulating factor, M-CSF, (100 ng ml^−1^, Miltenyi Biotec GmbH, Bergisch Gladbach, Germany) in α-minimum essential medium (MEM) (Invitrogen) for 7 days. The purity of BM-macrophage cultures was confirmed by FACS using CD11b (1:100, BD Biosciences, Mountain View, CA, USA) and F4/80 (1:100, BioLegend, San Diego, CA, USA) antibodies (average purity 80–90 %, not shown). After 7 days, BM-derived macrophages were cultured in α-MEM medium (Invitrogen) and differentiated to M1 and M2, respectively, with rIFNγ (20 ng/ml, Peprotech, USA) and rIL4 (20 ng/ml, R&D System, Minneapolis, MN, USA) for 48 h.

### Peritoneal macrophage cultures

Sterile 3 % thioglycolate (TG) was i.p. injected in C57BL/6 mice. After 5 days, mice were sacrificed, and peritoneal cells (Pec) were recovered by lavage with saline (S.A.L.F, Bergamo, Italy). Pec were centrifuged at 1200 rpm for 10 min at 4 °C. Supernatants were eliminated and pellets re-suspended in RPMI 1640 medium supplemented with penicillin (100 U/ml), streptomicin (100 U/ml), and ultra-glutamine (100 U/ml) (Lonza, Milano, Italy). Cells were counted with Turk’s solution (Merck Chemicals), plated at 5 × 10^6^ in 60-mm petri dishes (Becton Dickinson) and then incubated at 37 °C and 5 % CO^2^ for 1 h. Finally, cells were washed twice with saline and RPMI 1640 medium supplemented with 10 % FBS, penicillin (100 U/ml), streptomicin (100 U/ml), and ultra-glutamine (100 U/ml) was added. One hour after incubation at 37 °C and 5 % CO^2^, Pec polarization into M1 or M2 was performed by stimulation with rIFNγ (20 ng/ml, Peprotech, USA) plus LPS (100 ng/ml) for 4 h or rIL4 (20 ng/ml, R&D System, Minneapolis, MN, USA) for 18 h.

### RT-PCR analysis

RT-PCR analyses were performed on in vitro BM-derived macrophage assay and for IL4 gene therapy studies of the EAE model. Briefly, RNA was extracted using Trizol (Invitrogen) according to manufacturer’s instructions. Residual DNA was removed by treatment with 1 U DNase per 1 μg RNA (RQ1 RNase-free DNase, Promega) at 37 °C for 30 min. Complementary DNA (cDNA) synthesis from 3 to 5 μg total RNA was performed using Ready-To-Go You-Prime First-Strand Beads (Amersham) and Random Hexamer (New England Biolabs, Ipswich, MA, USA) according to the manufacturer’s instructions. Arg-1 (Mm00475988_m1), CCL17 (Mm005161 36_m1), CD206 (Mm00485148_m1), IFNγ (Mm01168134_m1), IL-1β (Mm01336189_m1), IL4 (Mm00445259_m1), IL6 (Mm00446190_m1), iNOS (Mm00440502_m1), TNF-α (Mm00443258_m1), and Ym1 (Mm00657889_mH). Messenger (mRNA) levels were measured by real-time RT-PCR (Applied Biosystems, Invitrogen). The 2−ΔΔCT method was used to calculate relative changes in gene expression [20].

### Fluorescence microscopy

BM-derived and Pec macrophages, differentiated as above, were stained for CD206 FITC (R&D) and, IL6 PE (Becton, Dickinson) for 5 min. Cells were then fixed in PFA 4 % for 10 min and incubated in 5 % FBS, 0.1 % Triton X-100, and PBS for 1 h to block any nonspecific binding site. Nuclei were stained with DAPI. A Leica SP5 (Leica Microsystems, Milano, Italy) confocal microscope and a GE Healthcare Delta Vision were used for image acquisitions.

### Co-culture of DCs-CD4^+^ T cells with M2 macrophages

BM cells were differentiated in dendritic cells (DCs) for 6 days in RPMI complete medium with GM-CSF (25 ng/ml) and rIL4 (25 ng/ml) (R&D Systems) and were activated with LPS (1 μg/ml) for 4 h. T cells were obtained from the spleens from TCR transgenic mice (2D2) specific for the myelin oligodendrocyte glycoprotein peptide, MOG 35–55 (Espikem, Florence, Italy), on the C57Bl/6 background [21] by preparing a cell suspension by mechanical dispersion using a cell strainer, followed by positive selection of CD4^+^ cell using Miltenyi columns (Miltenyi Italy). Fifty thousand activated DCs were co-cultured with 2 × 10^5^ CD4^+^ T cells and with the following concentration of MOG peptide: 0.3, 1, 3, 10 μM in triplicates. These cultures were added 2 × 10^5^ non stimulated (NS) or M2 macrophages, as indicated, and then incubated at 37 ° C and 5 % CO^2^ for 48 h.

### ELISA assay for mIL4, IL-2, and IL6

mIL2 and IL6 were measured from supernatants of DCs-CD4^+^ cells co-cultures with NS and M2 macrophages, by using a validated mouse-specific mouse IL2 and IL6 ELISA (R&D Systems). mIL4 was measured in supernatants and lysates of infected cells or CSFs (withdrawn from mice cisterna magna by capillarity) from IL4-injected EAE mice, using mouse IL4 ELISA (R&D Systems).

### Generation of mouse IL4-expressing lentivirus

Murine interleukin 4 (mIL4) was obtained from a plasmid expressing mIL4 in an adenoviral vector (Ad-G/IL4) [18]. The coding sequence of mIL4 was extracted with forward and reverse primers specifically designed to contain BamHI and SalI digestion sites, both synthesized by Primm S.r.l. (Milano, Italy). A third-generation lentivirus expressing mIL4 was generated cloning the mIL4 cDNA in the backbone of a p277 lentiviral transfer vector and producing a lentivirus as previously described [22]. A GFP-expressing lentivirus was also produced to be used as negative control in all the experiments.

### Mice

Six- to 8-week-old C57Bl/6 female mice were purchased from Charles River Laboratories (Calco, Italy). All mice were housed in specific pathogen-free conditions, in roomy cages, allowing free access to food and water. All efforts were made to minimize animal suffering and to reduce the number of mice used, in accordance with the European Communities Council Directive of November 24, 1986 (86/609/EEC). All procedures involving animals were performed according to the animal protocol guidelines prescribed by the Institutional Animal Care and Use Committee (IACUC # 449) at San Raffaele Scientific Institute (Milan, Italy). Because of the use of lentiviral vectors, animals were housed in isolated cages in the Biosafety Level 2 room of the Animal Care facility at San Raffaele Scientific Institute.

### IL4 gene therapy of EAE mouse model

Chronic EAE was induced in female C57BL/6 mice, by subcutaneous with 300 μl of 200 μg per mouse of MOG35–55 in Freund’s Adjuvant Incomplete liquid, IFA, (Sigma) supplemented with 8 mg ml^−1^*Mycobacterium tuberculosis* (strain H37Ra; Difco, Lawrence, KS, USA). Pertussis toxin (500 ng, List Biological Laboratories, Campbell, CA, USA) was injected i.v. on the day of the immunization and again 2 days later. IL4-expressing lentivirus or GFP-expressing lentivirus were injected in the cisterna magna (i.c.) of the mice at 12 d.p.i. A 30-gauge needle attached to a Hamilton syringe was inserted into the intrathecal space of the cisterna magna of anesthetized mice [23]. IL4-expressing or GFP-expressing lentiviruses (10 μl) in sterile phosphate-buffered saline (10^9^ PFU ml^−1^) were injected over 10 s. Mice were weighed and scored for clinical signs daily up to the day of culling. Clinical assessment of EAE was performed according to the following scoring criteria: 0 = healthy, 1 = limp tail, 2 = ataxia and/or paresis of hindlimbs, 3 = paralysis of hindlimbs and/or paresis of forelimbs, 4 = tetraparalysis, and 5 = moribund or death. EAE mice were killed at 34 d.p.i for real-time PCR and histological analysis.

### Preparation of CNS mononuclear cells

Mice were deeply anesthetized and perfused transcardially with cold phosphate-buffered saline at the indicated time point. The brains and the spinal cords were dissected out at the desired time point, removed, and homogenized through a 70-μm cell strainer in HBSS. Mononuclear cells were isolated using a neural dissociation kit (Milteny Biotech) and by 30/37/70 % Percoll (GE Healthcare) gradient centrifugation and collection of mononuclear cells from the 37/70 % interphase.

### CD11b^+^ cell separation

CNS mononuclear cells were spun at 300*g* for 10 min and then re-suspended in cold MACS buffer (1× PBS, 0.5 % BSA, 2 mM EDTA). Cells were incubated with biotin-conjugated monoclonal antibodies against CD11b (Mac-1, Rat IgG2b) (Myltenyi Biotech) at a concentration of 10 μl/10^7^ total cells in 40 μl MACS buffer for 10 min at 4 °C. Anti-biotin microbeads (20 μl/10^7^ cells in 80 μl MACS buffer) (Myltenyi Biotech) were added to the cells and incubated for 15 min at 4 °C. Finally, the cells were loaded on the MS-columns and the column-bound CD11b^+^ fraction isolated (Myltenyi Biotech). Cells were then centrifuged at 300*g* for 10 min, and re-suspended in 500 μl of TRizol (Invitrogen) and frozen at –80 °C.

### Histological evaluation

At 34 d.p.i., at least three mice per group were perfused through the left cardiac ventricle with saline plus EDTA 0.5 mM for 10 min followed by fixation with cold 4 % paraformaldehyde, PFA, (Sigma) in 0.1 M phosphate buffer (pH 7.4). Subsequently, the spinal cords and brains from EAE mice were carefully dissected out and post-fixed in 4 % PFA overnight and processed for cryogenic embedding. The quantification of neurological damage in EAE mice was performed via histological analysis of 10-μm frozen CNS sections of control or IL4-injected or GFP-injected EAE mice. Three different stainings were used to detect inflammatory infiltrates (hematoxylin and eosin), demyelination (Kluver Barrera), and axonal damage (Bielshowsky). Neuropathological findings were quantified on an average of 10 complete cross sections of spinal cord per mouse taken at eight different levels of the spinal cord. The number of perivascular inflammatory infiltrates were calculated and expressed as the numbers of inflammatory infiltrates per square millimeter, and demyelinated areas and axonal loss were expressed as percentage of damaged area.

### Immunohistochemistry and immunofluorescence

T cells were stained using a rat anti-CD3 (pan-T cell marker, Cat.No. MCA1477, Serotec, Oxford, UK), CD206 positive cells were stained with rat anti-mouse CD206 (Cat.No. MCA2235, Serotec, Oxford, UK), revealed with a biotin-labeled secondary anti-rat antibody (Cat.No. BA9400, Vector Laboratories, Burlingame, CA). Macrophages were stained with biotin-labeled BS-I isolectin B4 and IB4 (Cat.No. L2140-0, Sigma-Aldrich, St. Louis, MO). Biotin-labeled antibodies were developed with the ABC kit (Cat. No. PK-6200, Vector Laboratories, Burlingame, CA) followed by liquid DAB^+^ Substrate Chromogen System (Cat. No. K3467, Dako, Carpinteria, CA). We also stained sections with the same rat anti-mouse CD206 as above with a rabbit anti-mouse Iba1 (Dako, Carpinteria, CA) using fluorescent secondary antibodies as indicated. Nuclei were stained with DAPI. A Leica SP5 (Leica Microsystems, Milano, Italy) confocal microscope and a GE Healthcare Delta Vision were used for image acquisitions.

### Statistical analysis

Statistical evaluations of RT-PCR data, ELISA assay, EAE score, and immunohistochemical analyses results were expressed as mean ± s.d. or mean ± s.e.m, as appropriate. Results were analyzed using unpaired Student’s *t* test and Mann–Whitney *U* test for samples with unknown and potentially disparate variances. Analyses were performed using the Prism V5.0a software (Graph-Pad, San Diego, CA, USA). Statistical significance was ranked *P < 0.05, **P < 0.01, and ***P < 0.001.

## Results

### IL6 is expressed by IL4-polarized macrophages

**Fig. 1:**
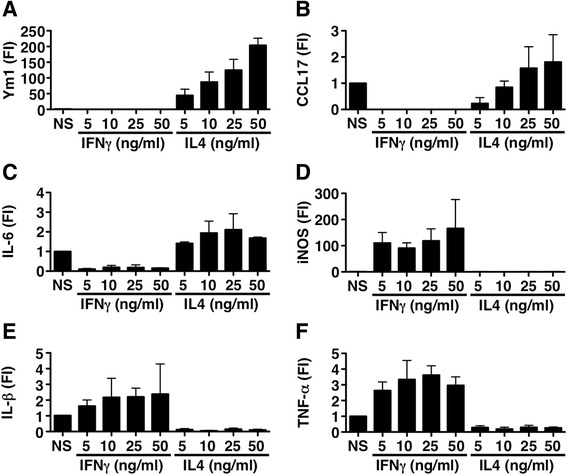
Gene expression of typical M1 and M2 markers in bone marrow-derived macrophages stimulated in vitro*.* **a**–**f** Real-time RT-PCR for Ym1, CCL17, IL6, iNos, IL-1β, and TNF-α, in macrophages non stimulated (NS) and stimulated with different doses of IFN-γ (M1) and IL4 (M2). Gapdh has been used as a housekeeping gene. Data are shown as fold induction (FI ± standard deviation) over NS

**Fig. 2:**
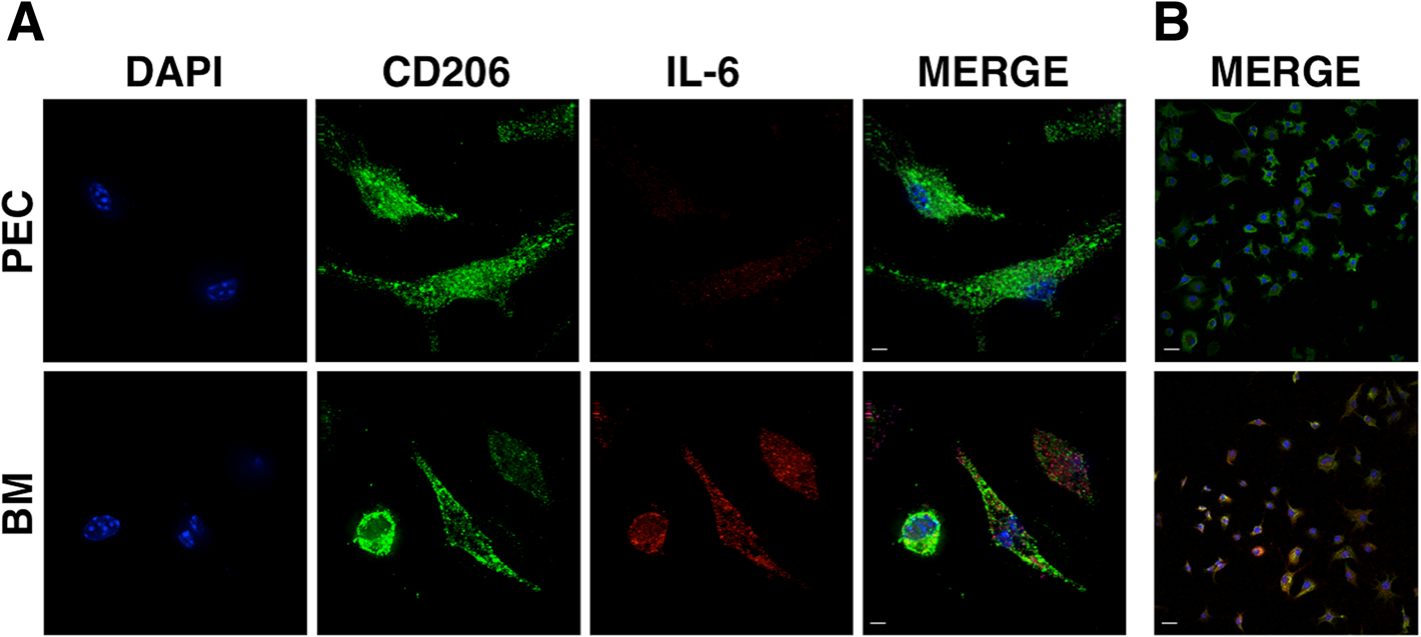
rIL4 induces IL6 expression in CD206^+^ macrophages. **a**, **b** Immunofluorescent staining for CD206 (*green*) and IL6 (*red*) reveals co-localization of IL6 and CD206 expression in bone marrow-derived (BM) macrophages, exposed to rIL4 at 20 ng/mL, but not in peritoneal (PEC) macrophages. **a** ×100, *scale bar* = 10 μm. **b** ×40, *scale bar* = 50 μm

We obtained bone marrow macrophages from C57BL/6 mice cultivated for 7 days in the presence of M-CSF. Cells where then polarized towards an M1 or M2 phenotype by using increasing concentrations of IFNγ or IL4, respectively. The expression of classical M2 markers such as Ym1 and CCL17 (Fig. 1a, b) was indeed confined to IL4-stimulated macrophages, but also, the pro-inflammatory cytokine IL6 was selectively expressed in M2 cells (Fig. 1c). IL6 expression was induced by IL4 also in peritoneal macrophages, although at much lower levels (Additional file 1: Figure S1). Expression of classical M1 markers such as iNOS, IL-1β, and TNF-α (Fig. 1d–f), were induced in IFN-γ-stimulated macrophages in a dose-dependent way. To verify if IL6 expression was indeed in the same cells bearing M2 markers and not in a subpopulation of macrophages with a different phenotype, we performed immunofluorescence on IL4-treated macrophages. We found co-expression of the M2 marker CD206 and IL6 in the same cells (Fig. 2).

### IL6-expressing macrophages are immunosuppressive

**Fig. 3:**
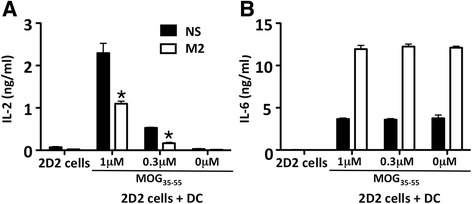
IL4-stimulated macrophages releasing IL6 are immune-regulatory. IL4-stimulated bone marrow-derived macrophages (M2) reduce IL-2 release from MOG-TCR transgenic T lymphocytes (2D2) stimulated with MOG_35–55_ (**a**), as compared to non stimulated macrophages (NS), while releasing high levels of IL6 (**b**) as measured by ELISA

We next asked if IL6-expressing M2 macrophages maintained the immunosuppressive functions associated to M2 macrophages. We stimulated T cells from 2D2 mice transgenic for the TCR specific for the myelin oligodendrocyte glycoprotein with MOG_35–55_, in the presence or in the absence of IL6 expressing M2 macrophages. As shown in Fig. 3, IL-2 release was significantly inhibited in the presence of IL6-expressing M2 macrophages (Fig. 3a), while IL6 was greatly increased (Fig. 3b). Thus, IL6-expressing M2 macrophages maintain their suppressive abilities.

### In vivo IL4 delivery to the inflamed CNS induces the expression of IL6 and M2 markers

**Fig. 4:**
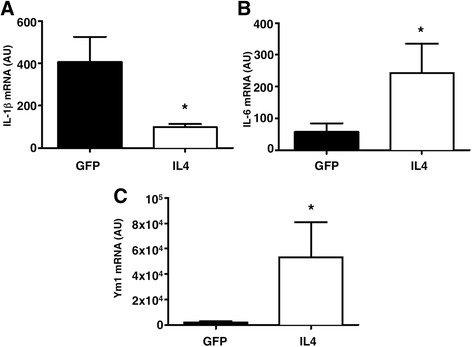
IL4 gene therapy induces M2 markers and IL6 expression in CNS-infiltrating CD11b^+^ cells in vivo. mRNA levels of IL6, IL-1β, and YM1 were measured by real-time RT-PCR in CD11b^+^ cells recovered from the CNS of EAE mice treated intracisternally with an IL4-expressing (IL4) or a GFP-expressing (GFP) lentivirus. IL4 gene therapy induces a decrease of IL-1β (**a**), and an increase of IL6 (**b**) and Ym1 (**c**), as compared to control-treated mice. *n* = 3 for each group. Gapdh has been used as a housekeeping gene. Data are shown as arbitrary units (AU ± standard deviation). *P < 0.05 (*t* test)

**Fig. 5:**
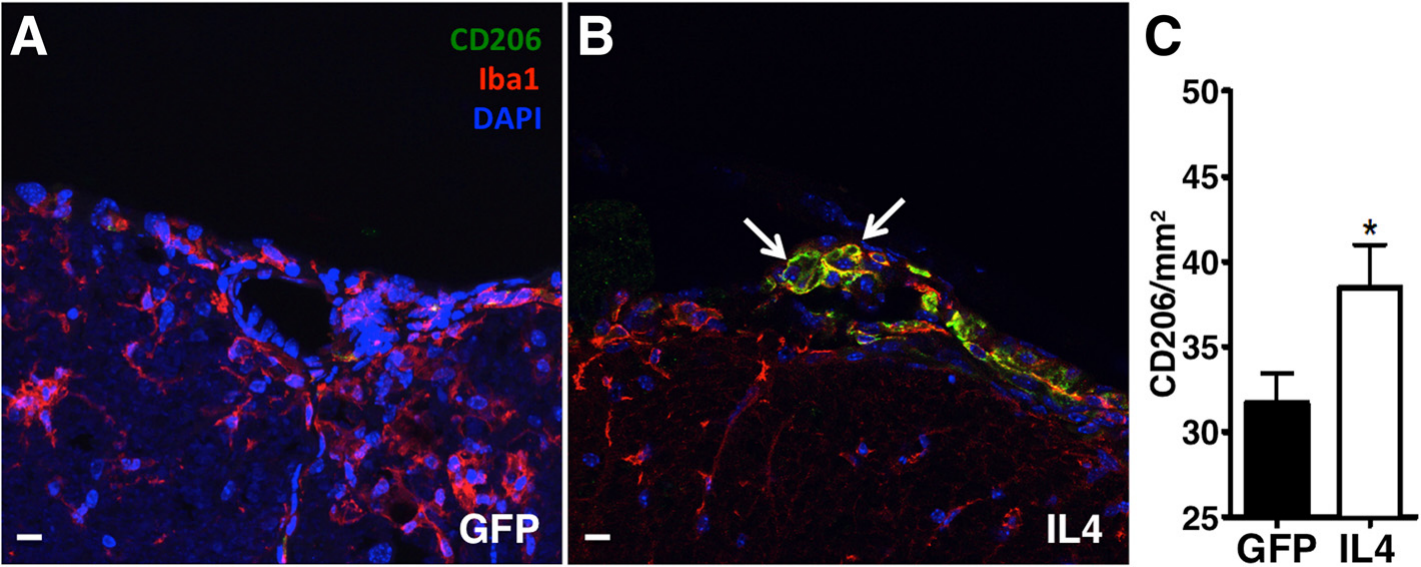
IL4 gene therapy upregulates CD206 in CNS-infiltrating macrophages of EAE mice. Immunofluorescence spinal cord sections of C57Bl/6 mice affected by EAE and treated, at 12 d.p.i, with the GFP-expressing (GFP, **a**) or the IL4-expressing lentivirus (IL4, **b**). The staining (CD206 in *green*, Iba1 in *red*, nuclei in *blue*, as indicated) highlights upregulation of CD206 in Iba1^+^ macrophages in EAE mice treated with IL4 gene therapy (**b**) as compared to mice treated with the GFP-expressing control virus (**a**). **a**, **b** ×40, *scale bar* 10 μm. Scale quantification of CD206^+^ macrophages in spinal cords is shown (± standard deviation) in (**c**). *P < 0.05 (*t* test)

To verify if IL6-expressing M2 macrophages play a role also during in vivo inflammatory reactions, we induced EAE in C57BL/6 mice by immunization with MOG_35–55_. We injected intracisternally an IL4-expressing lentiviral vector to induce the release of IL4 in the CSF of EAE mice. Control mice were injected with a GFP-expressing lentiviral vector. We found that CD11b^+^ myeloid cells (macrophages and microglia) purified from EAE mice treated with IL4 displayed reduced expression of the M1 marker IL-1β, increased expression of the M2 marker Ym1, and significantly increased expression of IL6, by RT-PCR (Fig. 4a–c). Indeed, the expression of the M2 marker CD206 was significantly increased in IL4-treated EAE mice (Fig. 5a–c).

### IL6-expressing IL4-treated EAE mice are protected from disease development

**Fig. 6:**
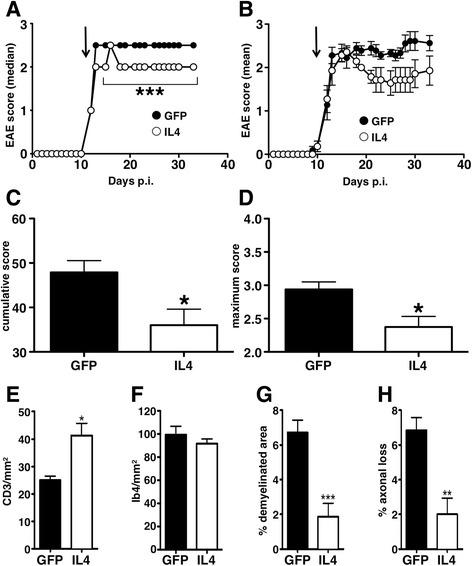
CNS IL4 gene therapy inhibits clinical and pathological signs of EAE. Clinical course of EAE mice intracisternally injected with the IL4-expressing (IL4, *open dots*) or the GFP-expressing (GFP, *closed dots*) lentivirus is shown either as median (**a**) or mean (±standard deviation) (**b**) values (EAE score is non-parametric). *Arrows* indicate the day of virus injection (day 12 post immunization). IL4-treated EAE mice are protected both in terms of clinical severity (including cumulative and maximum score, **a**–**d**), and demyelination (**g**) and axonal loss (**h**). The protective effect of IL4 gene therapy is associated to an increased number of infiltrating CD3^+^ T cells (**e**), but no modulation of IB4^+^ myeloid cells (**f**). ***P < 0.0001 (Mann–Whitney in **a**, *t* test in **g**); *P < 0.05 (Mann–Whitney in **c**, **d**, *t* test in **e**)

As previously described using different viral vectors [14, 15, 18], IL4 gene therapy of EAE mice, after disease onset, results in a significant decrease of clinical disease severity (Fig. 6a–d). Inhibition of EAE was confirmed also by neuropathological analysis showing a significant decrease in demyelination and axonal loss in IL4-treated mice (Fig. 6g–h). We confirm here that the protective effect of IL4 gene therapy in EAE is associated to a significant increase of infiltrating CD3^+^ T cells, while the number, but not the quality, of Ib4^+^ myeloid cells remains unaffected (Fig. 6e, f).

## Discussion

The role of innate immunity is under scrutiny in immune-mediated disorders because of its crucial role in initiating inflammation, regulating immune responses, and fostering tissue repair [2]. Investigating the role of monocytes, infiltrating and tissue-resident macrophages in immune-mediated disorders is complicated by the number of different functional phenotypes they can acquire [1, 2]. We replicated classical in vitro polarization assays on bone marrow-derived and peritoneal macrophages. We found, surprisingly, that IL6 is associated to M2 in bone marrow-derived macrophages and in peritoneal macrophages. In in vitro polarization experiments, macrophages usually assume extreme phenotypes, probably the only condition in which the M1/M2 paradigm holds partially true. IL6 had been already described associated to different M2 phenotypes, either M2b or M2c [1, 8, 9]. This is not surprising, since several groups have reported that macrophages in vivo usually display a blend of M1 and M2 markers, hypothesizing a spectrum of possible functional phenotypes [8, 9]. We asked the question if in our in vitro assays on bone marrow-derived macrophages, we were generating a mixture of functional phenotypes, one of them characterized by IL6 expression, or if IL6 was indeed expressed in cells bearing M2 markers. We found that IL6 was co-expressed, also at the protein level, by CD206^+^ M2 macrophages, and that these cells were immunosuppressive in vitro, despite releasing high levels of IL6. We have not further characterized in vitro the functional phenotype of these macrophages, namely M2a, M2b, and M2c, because of the ambiguity in the definition of these macrophage subtypes, mostly defined by the polarizing stimulus. Further, we are aware of the limitations of the in vitro suppressive assay. We therefore resolved to move to an in vivo setting, since we had already reported that induction of IL6 was associated to the anti-inflammatory effect of IL25 CNS gene therapy in a mild model of neuroinflammation [24]. We have repeatedly reported the therapeutic effect of IL4 CNS gene therapy in mouse and primate EAE [14–19]. Since IL4 is a classical M2-polarizing stimulus for macrophages, we asked the question if IL6 expression by infiltrating macrophages was associated to the protective effect of IL4 gene transfer. We found that CD11b^+^ cells purified from the brain of IL4-treated EAE mice displayed significantly increased expression of IL6, along with decreased levels of IL1β and increased levels of Ym1, thus a bona fide M2 phenotype, further confirmed by the increased levels of CD206 we found by immunofluorescence on infiltrating macrophages from IL4-treated EAE mice. We have currently similar findings in EAE mice successfully treated with IL27 CNS gene therapy (manuscript in preparation).

IL6 can signal through its classical receptor, a heterodimer composed by the cytokine-binding IL6R and by gp130 transducing the signal in the cell. IL6R expression is restricted to few cells in the CNS, while gp130 is ubiquitous [25]. gp130 can be activated to transduce its signal also by a complex of IL6 with the soluble form of its receptor, generated by proteolitic shedding or alternative splicing. This phenomenon, called trans-signaling, can target any gp130^+^ cell [26]. Outside the CNS, classical signaling is associated to IL6 protective effect, while pro-inflammatory activities of IL6 are mediated by trans-signaling [10]. IL6 signaling in the CNS has not been extensively studied, although trans-signaling appears to be associated with pro-inflammatory activities also in the brain [27–29]. Our findings suggest that in the CNS, however, classical IL6 signaling mediating protective, anti-inflammatory, signals may outweigh trans-signaling and explain the increased IL6 release associated to the protective effects of IL4 gene therapy on neuroinflammation. These results are contradictory with a long list of papers demonstrating the detrimental effects of IL6 during neuroinflammation. Among others, IL6 knock-out (k.o.) mice are resistant to EAE [30], CNS IL6 release redirects EAE inflammation to the sites of IL6 expression [31], IL6 inhibits the conversion of Th17 to Treg cells in the inflamed brain [32], and peripheral IL6 neutralization inhibits EAE [33]. It is not straightforward to reconcile these observations with our data. Most of these observations, however, rely on constitutive genetic modifications in mice, leading to possible constitutive alterations of the immune system in deletion mutants on one hand, to non physiological levels of the cytokine in IL6 overexpressing mice, on the other. In humans, monoclonal antibodies directed against IL6 are used in several chronic inflammatory diseases [10], including initial trials in neuromyelitis optica [34]. Multiple sclerosis development, however, has been reported in a patient under anti-IL6 treatment [35], leading to suspect a causal association [36]. Thus, targeting IL6 in the brain, as occurs for TNFα [37], may result in worsening of neuroinflammation, and suggests, rather, a prevailing protective effect of IL6 in the CNS.

The induction of IL6 in mice protected from EAE by IL4 gene therapy was not associated to a modulation in the number of IB4^+^ cells (i.e., macrophages and microglia). We confirm our previous finding that IL4 gene therapy of EAE is associated to an increase in infiltrating T cells that we previously described as being mostly Foxp3^+^ [18, 38].

## Conclusions

IL6 is mostly described as a pro-inflammatory cytokine, associated to the development of several inflammatory diseases. We found that IL6 is released by bone marrow-derived macrophages polarized with IL4 towards an anti-inflammatory, immune-regulatory, phenotype. Further, we found that IL6 expression was increased in infiltrating macrophages in mice protected from EAE by CNS IL4 gene delivery. We have not direct evidence of a protective role of IL6 per se; however, we think this data, together with others emerging from the literature, warrants a reconsideration of the significance of IL6 signaling during innate immune reactions in general, and during neuroinflammation in particular.

## Abbreviations

*CNS* central nervous system, *EAE* experimental autoimmune encephalomyelitis, *MOG* myelin oligodendrocyte glycoprotein, *p.i* post immunization, *PT* pertussis toxin

## Additional file

Additional file 1: Figure S1. Gene expression of typical M1 and M2 markers in peritoneal macrophages stimulated in vitro. (A–D) real-time RT-PCR for iNOS, Ym1, CCI17, and iL6, in non stimulated (NS), and in macrophages stimulated with different doses of iFn-γ (m1) and iL4 (M2). Gapdh has been used as a housekeeping gene. Data are shown as fold induction (Fi ± standard deviation) over NS. (TIF 42402 kb)
