# Supplementary figures and images for: IL4 induces IL6-producing M2 macrophages associated to inhibition of neuroinflammation in vitro and in vivo
Source: J Neuroinflammation. 2016 Jun 7;13:139. doi: 10.1186/s12974-016-0596-5 (PMC4895901; doi:10.1186/s12974-016-0596-5)

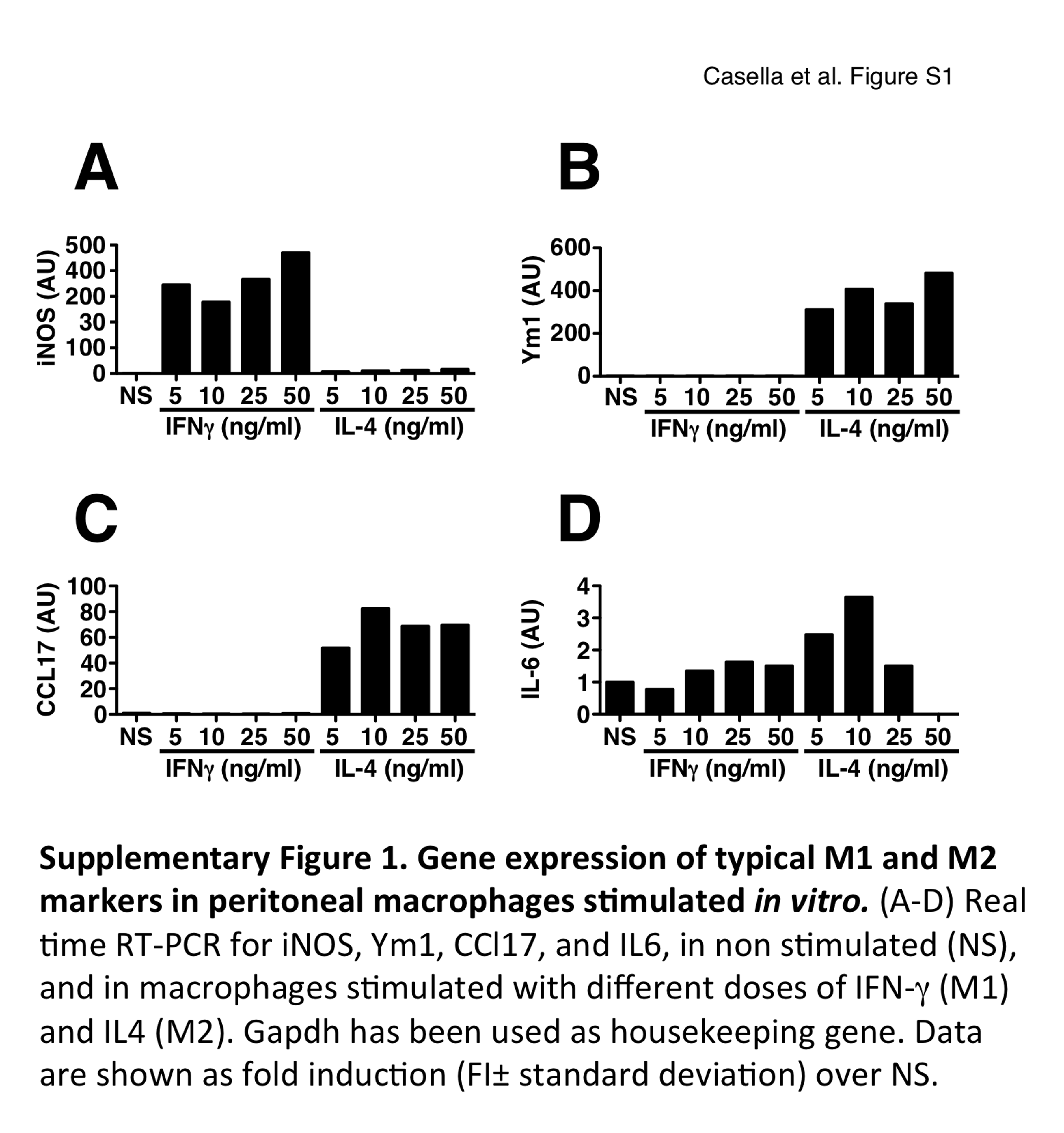

Supplement: Additional file 1: Figure S1. — Gene expression of typical M1 and M2 markers in peritoneal macrophages stimulated in vitro. (A–D) real-time RT-PCR for iNOS, Ym1, CCI17, and iL6, in non stimulated (NS), and in macrophages stimulated with different doses of iFn-γ (m1) and iL4 (M2). Gapdh has been used as a housekeeping gene. Data are shown as fold induction (Fi ± standard deviation) over NS. (TIF 42402 kb) [file 12974_2016_596_MOESM1_ESM.tif]
